# Assessment of knowledge about childhood autism spectrum disorder among healthcare workers in Makkah- Saudi Arabia

**DOI:** 10.12669/pjms.35.4.605

**Published:** 2019

**Authors:** Aalia Akhtar Hayat, Areej Habib Meny, Nabila Salahuddin, Faisal M.Alnemary, Kumar-Ricky Ahuja, Muhammad Waqar Azeem

**Affiliations:** 1 Dr. Aalia Akhtar Hayat, Maternity and Children Hospital, Makkah al Mukarama, Saudi Arabia; 2 Miss Areej Habib Meny, King Saud Bin Abdulaziz University for Health Science, Jeddah, Saudi Arabia; 3 Dr. Nabila Salahuddin**,** Mid-Essex Children’s Community Pediatric Medical Service, NHS Trust, United Kingdom; 4 Dr. Faisal M. Alnemary, Department of Special Education, Taif University, Taif, Saudi Arabia; 5 Dr. Kumar-Ricky Ahuja, Greenwich Community Pediatric Medical Services, London, United Kingdom; 6 Prof. Muhammad Waqar Azeem Department of Psychiatry, Sidra Medicine, Weill Cornell Medical College, Cornell University, Qatar

**Keywords:** ASD, ASD symptoms, Autism, Autism spectrum disorder, Healthcare professionals, Knowledge about autism, Neurodevelopmental disorders

## Abstract

**Objective:**

To measure the knowledge of healthcare professionals about increasingly prevalent Autism Spectrum Disorder (ASD) along with perceptions around its management and prognosis and comparison across various specialties.

**Methods:**

This Cross sectional survey based comparative analysis took place at Maternity and Children Hospital and King Faisal Hospital Makkah from December 2017 to May 2018. The validated self-administered “Knowledge about childhood autism among health workers” questionnaire was used along with additional questions regarding perceptions about ASD. The mean and mean percent scores were calculated. Chi squared test and ANOVA were applied to find the association between quantitative and qualitative variables respectively.

**Results:**

Out of 162 participants, 153 returned the questionnaire and 147 were included in final analysis. Physicians constituted 81.6% (120) of participants. The mean score for participants was 9.80(S.E.M ±0.32) where non-physicians yielded higher mean score (11.2±4.41) as compared to physicians (9.6±3.28) (p=0.113). Psychiatrists had highest score of 16/19 while general physicians had lowest (6/19). Participants with more years of experience had higher mean scores (p-value = 0.01). About 72.10% (106) of participants opted for medication as a treatment option. Nearly 38.1% (56) of participants were skeptical about improvement of ASD with early interventions.

**Conclusion:**

There is a lack of knowledge about ASD amongst healthcare professionals in Saudi Arabia. Experienced professionals working with ASD children can be utilized to deliver targeted trainings nationwide.

## INTRODUCTION

One in every 68 children are estimated to have autism spectrum disorder (ASD), making it one of the most common neurodevelopmental disorders.^1^ ASD is marked by deficiencies in communication, behaviors and social interaction skills that may have negative impact on people with autism and their caregivers.^2^ In Arab Gulf countries, prevalence ranging between 1.4-29/10,000 has been reported.^3^

There is no known cure for ASD, but it is established that early intervention and intensive behavioral therapy (BT) is concomitant with a better outcome.^4^,^5^ However, the average time taken by a child to reach specialist services for early intervention is too long, especially in countries like Saudi Arabia (SA) and this causes unnecessary delay in early detection and intervention, compromising a productive outcome.^4^,^6^ One major cause of this delay was found to be limited level of knowledge and awareness amongst healthcare professionals (HCPs) about symptoms and presentations of ASD.^7^ Various studies around the globe yielded heterogeneous results regarding level of awareness about ASD amongst a variety of health professionals ranging from various specialties of physicians to a variety of non-physicians professionals.^7^,^8^ By increasing the awareness and knowledge about this disorder, prompt referral and early intervention can be result in shorter waiting times thus increasing chances of a better prognosis.^4^,^5^ Hence the purpose of this survey was to assess knowledge and perceptions about ASD among a variety of HCPs in Makkah, Saudi Arabia and to provide useful suggestions in the light of study outcomes.

## METHODS

Ethical approval was obtained from the Ethical committee of the Hospital. Informed consent was taken after study objectives were explained and then the survey was distributed. Respondents were requested to complete and return it there and then, in order to ensure a good response rate and decreased chances of obtaining information from collateral sources.

Data were collected by convenience sampling using a survey form encompassing demographic details, a validated questionnaire (English) titled Knowledge about Childhood Autism Among Health Workers (KCAHW)^8^,^9^ was used. Data was also collected regarding management preferences, prognosis of ASD, and myths and misconceptions, since there are no previous studies that took into account local health belief model.^10^

KCAHW Questionnaire is self-administered and contains 19 items, each having three options to choose from (correct option yields 1 score while incorrect ones get a 0). It has four domains. Domain-1 consists of eight items about difficulties in social interaction. Domain-2 has one item about communication and language development. Domain-3 contains four items about restricted, repetitive and stereotyped behaviors and interests. Domain-4 contains six items about etiology, comorbid conditions and the onset. A maximum score of 19 and minimum score of 0 is achievable.

Data was entered in SPSS 21.0 version after scoring the responses. Descriptive analysis was done to calculate means, frequencies and percentages. Mean and SD of the whole sample along with mean percent score were calculated. Comparison of mean scores of physician vs non-physicians was done by applying t-test keeping value of p < 0.05 (confidence interval 95%). Chi squared test was applied to observe association between qualitative variables like gender and nationality etc.

## RESULTS

The total number of participants was 162. Questionnaires filled and returned totaled 153, yielding a response rate of 94%. Out of these, six had incomplete information and were excluded from the final analysis resulting in an inclusion of 147 questionnaires. Of these, 113 participants completed the KCAHW Questionnaire (rest did not respond for one or two items so were not included for mean score but included in individual analysis)

Males covered 44.90% (66) of the study sample and females constituted 48.30% (71). About 6.8% were unidentified. Mean age of the sample was 28.02 (SD ±13.09). About two-thirds (104) of the participants were Saudi Nationals whereas 6.80% did not reveal their nationality.

Physicians constituted 81.6% (120) of the sample. HCPs consisted of pediatricians, psychiatrists, psychologists, physiotherapists, occupational therapists (OTs), general physicians (GPs), surgeons, gynecologists, medical specialists and few medical trainees.

Pediatricians were highest in number (23.80% =35) and physiotherapists comprised 14.3% (21) of the total (highest number among non-physicians). Ratio of participants from the two hospitals was 3:2. Less than half (40.8%=60) had a clinical experience of more than 5 years. The total score of the study population was 1108/2147 with the mean score 9.80(S.E.M ±0.32). Mean percent score was found out to be 51.60%.

The mean score in Domain-1 (communication and social interaction) was 5.7/8 (S.E.M ±0.14) (71.25%). In Domain-2 (language), it was 0.62/1(S.E.M ±0.04) (62%). In Domain-3 (behaviors) it was 2.2/4(S.E.M ±0.11)) (55%) and in Domain-4 (etiology), it was 0.73/6 (S.E.M ±0.06) (12.16%). Males had mean score of 9.8(S.D ±3.32) vs 9.7(S.D±3.72) amongst females. Saudi nationals scored 9.5(S.D ±3.54) as compared to 10.6(S.D ±3.28) by other nationalities.

The mean score for physicians’ and non-physicians was 9.6(S.D ±3.28) and 11.2(S.D ±4.41) respectively. On applying independent sample t test, difference between the two groups was not statistically significant (p=0.225). Compared among specialties, using ANOVA, difference between mean scores was statistically significant (p= 0.012). Psychiatrists had highest score of 16/19 (n=1) while general physicians had lowest score of 6 (n=1) (Table-I).

**Table I:** Mean scores of health professionals on KAHCW.

Specialty	N	Mean	Standard deviation	Minimum score	Maximum score
Pediatrics	28	9.96	3.73	1.00	16.00
Psychiatry	1	16.00	-	16.00	16.00
Psychology	4	14.50	1.73	13.0	16.00
Physiotherapy	7	8.71	4.07	2.00	12.00
Occupational therapy	1	9.0	-	9.00	9.00
General physician	1	6.0	-	6.00	6.00
Resident	2	15.50	0.70	15.00	16.00
Surgery	15	9.46	2.82	5.00	14.00
Gynecology	28	9.86	2.86	3.00	16.00
Internal medicine	26	8.84	3.29	3.00	16.00

Total	113	9.80	3.44	1.00	16.00

There was statistically significant difference (p=0.01) in knowledge among the professionals based upon their years of clinical experience. Participants with < 1 year of clinical experience had score of 8.22, with 1-5years experience had a score of 9.5 and > 5 years of experience had 10.9 mean score.

Participants were found to have minimum knowledge about symptoms of ASD such as staring purposelessly and preoccupation with parts of objects, along with etiology and comorbidities associated commonly with the condition. They scored better regarding communication and social behavior symptoms. Table-II.

**Table II:** Components of KCAHW yielding maximum and minimum scores on knowledge.

Areas of KCAHW yielding maximum score on knowledge	Yes	No	Do not know	Did not respond	Areas of KCAHW yielding minimum scores on knowledge	Yes	No	Do not know	Did not respond
1. Failure to develop peer relationships	85.70%	1.40%	12.90%	-	1. Autism can be associated with epilepsy	28.60%	40.00%	29.90%	1.40%
2. Impaired non-verbal behaviors	83.00%	1.40%	13.60%	2.00%	2. Persistent preoccupation with parts of objects	35.4%	6.80%	44.2%	13.6%
3. Lack of shared interests or activities	79.60%	7.50%	11.60%	1.40%	3. Autism can be associated with mental retardation	44.20%	29.30%	25.90%	0.70%
4. Loss of interest in environment	73.50%	21.00%	10.90%	0.70%	4. Autism is neurodevelopmental disorder	46.90%	29.305	23.10%	0.70%
5. Lack of social/emotional reciprocity	72.10%	6.80%	20.40%	0.70%	5. Staring into open space without focus	49.00%	25.90%	24.50%	0.70%

In the question about interventions helpful in the management of ASD, a vast majority of professionals (72.10%=106) opted for medication as a treatment option. There was no statistically significant difference between physicians and non-physicians in choice of medication as a treatment option (P=0.31).

Regarding non-pharmacological treatment options, 57.8% (85) thought a referral should be made to psychiatrists, 56.5% (83) thought a referral to psychologists is important, 68.7% (101) believed in referring to the speech therapists (STs) whereas 51.7% (n=76) thought of occupational therapy (OT) as a possible intervention. However, 65.65% (n=21) of medical specialists, 50% (n=16) of gynecologists, 38.14% (n=13) of pediatricians, 33.33% (n=12) of surgeons and 25.00% (n=1) of psychologists did not consider OT as a useful intervention in management. The difference between physicians and non-physicians regarding myths and misconception about ASD was not significant statistically Table-III.

**Table III:** Response of participants regarding myths and misconceptions in ASD.

	Agree	Not sure	Disagree	P-value (comparison of physician and non-physicians response)
1. ASD is a rare condition as compared to the West.	12.9%	43.5%	40.8%	0.42
2. There is little awareness regarding ASD amongst health professionals in Saudi Arabia.	75.55%	17.75%	5.45%	0.10
3. Children with ASD do not show social attachment even to parents	38.1%	28.6%	31.3%	0.86
4. Even with early interventions, ASD has poor prognosis	14.3%	23.8%	59.9%	0.73
5. Enough campaigns have been done to raise awareness about ASD in this country.	19%	29.3%	49.0%	0.22
6. Vaccination is a cause of ASD.	4.8%	21.8%	69.4%	0.99

## DISCUSSION

This study aimed at exploring HCPs’ knowledge regarding ASD in Saudi Arabia. Preferred approaches towards management, pharmacological and otherwise were also looked into. A few questions regarding misconceptions and awareness were incorporated in order to make a comprehensive assessment regarding the understanding and concepts of HCPs around this important neurodevelopmental disorder.

Mean score of all healthcare professionals was 9.80±3.44. This translates into a mean percent of 51.57 and implies that HCPs in our study had moderately low levels of knowledge regarding childhood ASD overall. This is comparable to the scores in previous studies done in community health workers that yielded scores of 9.6 and 9.8.^9^

However, mean percent improved from 51.57% to 65.53% when only domains 1, 2 and 3 (questions about sign and symptoms of ASD) were taken into account, implying that HCPs had somewhat better understanding of presentation of ASD as compared to the etiology or comorbidities addressed in domain 4 of KCAHW. Similarly, Eseigbe et al found the highest knowledge gap was associated with onset of ASD and its comorbidities and the least was concerning communication impairments.^7^

Regarding individual domains like 1 (social interaction), 2 (communication) and 3 (repetitive and odd behaviors), mean percent was 71.25%, 62.00% and 55% respectively. Poorest knowledge was about comorbidities and etiology of ASD (domain 4) i.e. 12.16%. Out of HCPs only 28.60% and 44.20% knew that ASD can be a comorbidity associated with epilepsy and intellectual disability (ID) respectively (Table-III).

This has practical implications as more than 50% children with ASD might have a comorbidity with ID, and in some studies prevalence of ID in ASD is reported as high as 72.8%.^11^ This may present first time to non-mental HCPs and limited knowledge about this might cause significant delay in early detection and referral. This needs to be addressed as areas of significant knowledge gaps have been identified through our study, which need to be filled with specific and directed education and training.

Our findings are somewhat similar with an African study which found pediatricians and psychiatrists practicing in a tertiary health facility were associated with having higher knowledge about ASD as compared to general practitioners.^7^

Similarly, in our study amongst clinicians, psychiatrists followed by pediatricians had highest knowledge. Another similar study about HCPs in tertiary care centers (psychiatry or pediatrics) found the mean knowledge to be 12.35±4.4, which was higher than in our study.^12^ Possible reasons could be that the HCPs were recruited from tertiary care centers dealing with either children or with mental health issues and had understandably higher knowledge regarding this childhood developmental disorder as compared to our HCPs which were working in either a tertiary hospital with no pediatrics or psychiatric services or in a children hospital with newly established child psychiatry and pervasive developmental disorder services.

A positive finding was that medical trainees had a high score of 81.58%. This might be in agreement with previous studies that medical curriculum and psychiatric clerkships do equip students with an ample knowledge about psychiatric illnesses however there may be fading away of this knowledge without ongoing training and education.^13^

Interestingly, pediatricians had a mean score of 9.96, higher only by fractions than other specialties like gynecology (9.80) and surgery (9.46). This may have important implications as pediatrics and general practice are the specialties that are the first point of contact for these children and families. Running programs and workshops to sensitize these specialties may prove to have an exponential effect in successful early detection and prompt referral of such children.

In addition to the KCAHW questionnaire, we asked a few questions to assess the professionals’ views about modalities of interventions needed to manage ASD, as this would affect referral to appropriate and concerned professionals. Participants had to choose one or more options out of various pharmacological and non-pharmacological measures (like referral to speech therapy (SP), OT and BT which they deemed would help in management of ASD.

Most of the HCPs i.e. 72.10% (106) opted for medication as a treatment option. Interestingly, there was no statistically significant difference between physicians and non-physicians in this regard (P=0.31). Keeping in view that so far, no medications have been proven to be efficacious in the treatment of the core social communication impairment seen in ASD and current pharmacological management (mostly with risperidone and aripiprazole) is only aimed at targeting symptom of irritability associated with ASD.^14^ Having said that however, in some children, the usage of SSRIs for certain repetitive and stereotypic behaviors as part of the main behavioral and rehabilitative program can be very useful.^15^

Regarding non-pharmacological treatment options, only near half of HCPs believed in referring to speech therapists, psychiatrists, psychologists and occupational therapist as a possible intervention. Alarmingly, many did not recognize the key role played by psychiatrists and psychologists in the diagnosis and management of ASD. This is even higher than previous studies, which showed that more than 25% of HCP’S did not consider the need for ST and special education services in the management of this disorder.^8^ Disturbingly, 65.65% of medical specialists, 50% of gynecologists, 38.14% of pediatricians, 33.33% of surgeons and 25.00% of psychologists did not consider occupation therapy as a useful intervention in management of ASD. This is in line with previous research in Saudi Arabia which has highlighted decreased awareness and knowledge about importance of OT in rehabilitation.^16^

There were many misconceptions regarding etiology, symptomatology and prognosis of ASD. Overall, no statistical difference was found between physicians and non-physicians regarding questions related to myths and misconceptions about ASD. In response to the notion that “ASD is a rare condition as compared to the West”, although 60% disagreed, a large number of professionals were either not sure or thought otherwise regarding the increasing autism prevalence. Without having prior knowledge and awareness we cannot expect HCP’s to be prepared to deal with the rising magnitude of this condition.

One popular belief is that vaccinations can cause ASD.^17^ Interestingly although the majority of HCP’s (70%) disagreed with this notion, there was still one third of HCP’s who were doubtful about this misconception. Nearly 14% of pediatricians were either not sure about this notion or agreed that vaccines can cause autism. This is an important area that needs to be addressed. This may hamper the routine process of vaccination for children and a resultant rise in infectious diseases.^18^ Another important misconception about children with ASD was that they do not show social attachment even to parents. Only one third of HCP’s had understanding that children with autism may have social attachments with caregivers and parents.^19^ Two thirds either denied or admitted not having knowledge about this. This can impede the early diagnosis and referral of children with ASD who show attachment to the primary caregivers, thus increasing the service delivery latency.

While exploring views “Even with early interventions, ASD has poor prognosis”, 38.1% disagreed or weren’t sure that children with ASD can improve. This was the most worrisome finding as early intervention has a proven role in improving functionality and outcome in autism.^4^

Finally, two questions were asked in order to view the participants’ perceptions about their own knowledge and understanding around ASD along with their views about awareness activities and campaigns which have occurred. More than three quarter of HCP’s agreed to the notion that “There is a little awareness about ASD among HCPs in Saudi Arabia”, whereas half of them were not sure or thought otherwise about the statement that “Enough campaigns had been done to raise awareness about autism in the Country”. This could be because of most of the courses and symposiums about autism have been carried out in specialist settings that provide services for people with autism or special needs. Thus, this may limit the abilities of HCPs like pediatricians and surgeons to attend such courses.

### Limitations of the study

Our sample was not fully representative, as it was recruited from a tertiary care government hospital and from maternal and children hospital in Makkah. Thus, results cannot be generalized to the whole region or country. In addition, the numbers in each specialty were not equal which can give an uneven perspective.

Despite these limitations, our study is the first of its kind in Saudi Arabia, as it does not only provide evidence and comparison about presence of a huge knowledge gap regarding this prevalent disorder amongst various physicians and non-physicians, but also identifies the specific areas of knowledge deficiency. Decreased knowledge about comorbidities associated with ASD, prognosis and possible treatment modalities especially non-pharmacological options, can be improved by stressing around these areas, as well as improving education and training, inclusion in discussions and ward rounds along with use of media and electronics in order to increase awareness and early recognition of autism amongst healthcare professionals countrywide.

## CONCLUSION

There is a dearth of knowledge about ASD amongst HCPs in Saudi Arabia. Specific and focused strategies need to be put on in order to bridge this knowledge gap to ensure early detection, timely referral, appropriate diagnosis and evidence based interventions. Experienced HCPs working with children with ASD can be deployed in order to deliver tailored trainings especially regarding comorbidities and appropriate referral modalities to HCPs nationwide.
